# Development and Validation of a High-Performance Liquid Chromatography Diode Array Detector Method to Measure Seven Neonicotinoids in Wheat

**DOI:** 10.3390/foods13142235

**Published:** 2024-07-16

**Authors:** Serenella Seccia, Stefania Albrizio, Elena Morelli, Irene Dini

**Affiliations:** Department of Pharmacy, University of Naples Federico II, Via Domenico Montesano 49, 80131 Napoli, Italy; seccia@unina.it (S.S.); salbrizi@unina.it (S.A.); emorelli@unina.it (E.M.)

**Keywords:** pesticide residue analysis, SPE-HPLC/DAD, food security, insecticides, neonicotinoids, cereals, acetamiprid, clothianidin, dinotefuran, imidacloprid, nitenpyram, thiacloprid, thiamethoxan

## Abstract

Neonicotinoids (NEOs), used as insecticides against aphids, whiteflies, lepidopterans, and beetles, have numerous detrimental impacts on human health, including chronic illnesses, cancer, infertility, and birth anomalies. Monitoring the residues in food products is necessary to guarantee public health and ecological balance. The present work validated a new method to measure seven neonicotinoid insecticides (acetamiprid ACT, clothianidin CLT, dinotefuran DNT, imidacloprid IMD, nitenpyram NTP, thiacloprid TCP, and thiamethoxan THT) in wheat. The analytical procedure was based on simple and fast wheat sample cleanup using solid-phase extraction (SPE) to remove interferents and enrich the NEOs, alongside the NEOs’ separation and quantification by reverse-phase chromatography coupled with a diode array detector (DAD). The validation process was validated using the accuracy profile strategy, a straightforward decision tool based on the measure of the total error (bias plus standard deviation) of the method. Our results proved that, in the future, at least 95% of the results obtained with the proposed method would fall within the ±15% acceptance limits. The test’s cost-effectiveness, rapidity, and simplicity suggest its use for determining the levels of acetamiprid, clothianidin, dinotefuran, imidacloprid, nitenpyram, thiacloprid, and thiamethoxam in routine analyses of wheat.

## 1. Introduction

Over the past few decades, European strategies have been directed towards diminishing pesticide utilization and promoting more sustainable farming practices. The Common Agricultural Policy in 1962 and the European Directive 2009/128/EC issued by the European Commission in 2009 established an Integrated Pest Management System and advocated for reducing reliance on agricultural pesticides [1]. Despite these measures, the practical implementation of these policies has not successfully achieved its intended objectives. The insecticide market is continually expanding. In 2021, the worldwide insecticide market was valued at USD 14 billion. This market is projected to grow at a compound annual growth rate (CAGR) of 5% from 2022 to 2030, reaching an anticipated value of USD 22 billion by the end of the forecast period [2]. Neonicotinoids (NEOs), constituting more than a quarter of the global market, were forecasted to attain an annual market worth close to USD 10 billion by 2025 [3]. This success is because neonicotinoids demonstrate superior precision in pest management than traditional insecticides, and they do not exhibit cross-resistance, enabling them to effectively control pests which have developed resistance to earlier generations of insecticides [4]. Neonicotinoids negatively impact ecosystems, subterranean and surface water resources, and food security by influencing the relationship between crop yields and the death rate of natural pollinators, such as bees, which support the food sector through sustainable farming practices [5]. Moreover, they accumulate in the human body [6]. Prolonged exposure to NEOs has been linked to a variety of adverse effects on human health, such as neurological toxicity (autism spectrum disorders, muscle tremors, and memory loss) [7], hepatotoxicity [8], diabetes, acute respiratory and cardiovascular events [9,10], and dysfunctions in growth, reproduction [11], and immunity [12]. Neonicotinoids are categorized into three distinct generations. The initial generation comprises chloropyridinyl compounds: acetamiprid, imidacloprid, nitenpyram, and thiacloprid. The second generation consists of chlorothiazolyl compounds, specifically clothianidin and thiamethoxam. The third generation includes tetrahydrofuryl compounds, such as dinotefuran. Other neonicotinoids have been introduced, including cycloxaprid, guadipyr, flonicamid, flupyradifurone, imidaclothiz, paichongding, and sulfoxaflor. Seven neonicotinoids are available worldwide: acetamiprid, clothianidin, dinotefuran, imidacloprid, nitenpyram, and thiacloprid [13]. Unfortunately, they can persist in the environment under natural conditions from several months to years, and, sometimes, their metabolites have comparable or even higher toxicity [14]. A lot of analytical approaches have been developed to measure NEOs. These include chromatography techniques (high-performance liquid chromatography coupled with ultraviolet, diode array, or mass spectrometry detectors) [15], immunoassays [16], or sensors (optical and electrochemical) [17,18]. Typically, a sample pretreatment is performed to concentrate the NEOs and eliminate the matrix interferences before instrument detection [13]. The pretreatment methods include solvent extraction methods such as SLE (solid–liquid extraction) [19], LLE (liquid–liquid extraction) [20], SPE (solid-phase extraction) [21], and the QuEChERS (Quick, Easy, Cheap, Effective, Rugged, and Safe) method [22]. Additionally, there are novel miniaturized and automated methods such as LLME (liquid–liquid microextraction) [23], dSPE (dispersive solid-phase extraction) [24], MSPE (magnetic solid-phase extraction) [25], SPME (solid-phase microextraction) [26], and matrix solid-phase dispersion (MSPD) [27]. In this work, an analytical method that employs a cleanup procedure combined with reverse-phase HPLC-DAD was developed and validated for the determination of seven NEOs—acetamiprid (ACT), clothianidin (CLT), dinotefuran (DTN), imidacloprid (IMD), nitenpyram (NTP), thiacloprid (TCP), and thiamethoxan (THT)—in wheat since the World Health Organization and the Food and Agriculture Organization established an estimated acceptable daily intake for them [28]. To this end, in the hope of improving the recovery of NEOs, the performance of a new polymer system (STRATA XPRO) was compared with the QuEChERS method typically used to optimize the recovery of NEOs from food matrices. Finally, the entire analytical procedure was validated using the accuracy profile strategy, a strategy proposed by the Commission of the Société Française des Sciences et Techniques Pharmaceutiques (SFSTP) for validating quantitative analytical procedures [28,29].

## 2. Materials and Methods

### 2.1. Solvents and Reagents

Acetonitrile (ACN) for HPLC, methanol (MeOH), and dichloromethane for pesticides were purchased from Carlo Erba Reagents (Carlo Erba Reagents, Cornaredo, MI, Italy). Formic acid (HPLC grade) was bought from Sigma-Aldrich (Sigma-Aldrich, St. Louis, MO, USA).

Water for HPLC was obtained by distilling and vacuum-filtering water using MILLIPORE filters (Merck, Burlington, VT, USA).

Reagent-grade anhydrous magnesium sulfate (MgSO_4_) and sodium chloride (NaCl) were obtained from Merck (Darmstadt, Germany), and activated primary secondary amine (PSA) and graphitized carbon black (GCB) adsorbent were obtained from Supelco (Bellefonte, PA, USA). STRATA X PRO^®^ prepacked cartridges (100 mg/6 mL) were purchased from Phenomenex (Phenomenex, Torrance, CA, USA).

### 2.2. Analytical Standards

Acetamiprid, clothianidin, dinotefuran, imidacloprid, nitenpyram, thiacloprid, and thiamethoxam (PESTANAL^®^) were bought from SIGMA-ALDRICH (St. Louis, MO, USA).

### 2.3. Sample Preparation

The wheat samples, consisting of ground and sieved kernels (2 mm), were purchased from local supermarkets in Naples, Italy. The samples thus prepared were stored at 4 °C in glass bottles. All analyses were conducted in triplicate. 

#### 2.3.1. Sample Extraction and Cleanup with the QuEChERS Method

In a 50 mL screw-top centrifuge tube (Falcon^®^; Fisher scientific, Hampton, NH, USA), water (5 mL) and acetonitrile (10 mL) were added to aliquots of the ground and sieved sample (1 g). The sample was left to rest for 5 min, sonicated in an ultrasonic bath for 15 min, and mixed in a vortex for one minute. MgSO_4_ (4 g) and NaCl (1 g) were successfully added, and the sample was vortexed for 1 min. The extracts were centrifuged at 6000 rpm (10 min) twice. Aliquots of 1.5 mL of the top layer were added to a 2 mL centrifuge tube containing 150 mg MgSO_4_ + 50 mg PSA + 50 mg GCB, again vortexed and centrifuged at 6000 rpm. The extracts were collected, concentrated to 1 mL, and injected into HPLC.

#### 2.3.2. Sample Extraction and Cleanup with STRATA X PRO Cartridges

In a 50 mL screw-top centrifuge tube (Falcon^®^), 4 mL of water was added to aliquots of the sample (1 g). The sample was left to rest for 5 min; then, acetonitrile (2 mL) was added, sonicated in an ultrasonic bath for 5 min, vortexed for 2 min, and centrifuged at 6000 rpm for 10 min (twice). The supernatant was recovered and concentrated to 3 mL using a Rotavapor. Successively, the sample was loaded onto a prepacked STRATA X PRO^®^ cartridge (Phenomenex, Torrance, CA, USA). The samples were eluted with 10 mL (5 × 2 mL) of dichloromethane containing 10% methanol. The extracts were collected, concentrated to 1 mL, and injected into HPLC. The extracts were evaporated, dissolved into 1 mL with CH_3_OH, and injected into HPLC.

### 2.4. HPLC (High-Performance Liquid Chromatography)’s Parameters

HPLC experiments were performed on an Agilent Technologies 1200 series system interfaced with a diode array detector, setting the wavelength to 260 nm. The analytical column was a Kinetex C18 (150 mm × 4.6 mm ID, 5 μm particle size) from Phenomenex (Torrance, CA, USA), with a Phenomenex C18 guard cartridge (4 × 8 mm). The injection volume was 20 μL, and the flow rate was 1 mL/min. The mobile phase was a gradient of two eluents: Eluent A (water containing 0.2% formic acid, distilled and vacuum-filtered on MILLIPORE filters) and Eluent B (acetonitrile). The gradient was obtained by increasing the B content to 15% in the first 5 min, 80% in 15 min, and maintaining this ratio for 20 min. The NEOs’ retention times are reported in Table 1.

### 2.5. Method Validation

Method validation followed the French Society of Pharmaceutical Sciences and Techniques (SFSTP) guidelines, using the total error approach (accuracy profile) [28,29]. The predefined acceptance limit was ±15%, and the *β*-expectation tolerance interval was 95%.

#### 2.5.1. Experimental Designs

The validation and calibration designs both consisted of 3 days (*k* = 3), 3 replicates (*n* = 3), and 3 levels (*m* = 3) of concentration, totaling 27 trials. This number was chosen as a reasonable compromise between the number of analyses that could be completed in one day and the cost of the validation study.

A stock solution (0.8 mg/mL) was prepared for each insecticide by dissolving 40 mg of each analyte in 50 mL of acetonitrile. 

A multicomponent standard solution (10 μg/mL) was prepared by diluting each stock solution with the initial mobile phase.

#### 2.5.2. Calibration Standards

Calibration standards were prepared by diluting appropriate volumes of the multicomponent solution with the initial mobile phase to obtain three concentration levels: 0.01, 0.05, and 0.1 μg/mL.

Solutions were kept in darkness at 4 °C and protected from light (a condition in which they are stable for 3 months).

### 2.6. Statistical Analysis

Microsoft Office Excel 2010 (Microsoft, Redmond, WA, USA) was used to perform statistical analyses.

## 3. Results

### 3.1. Cleanup Procedure Optimization

**Figure 1:**
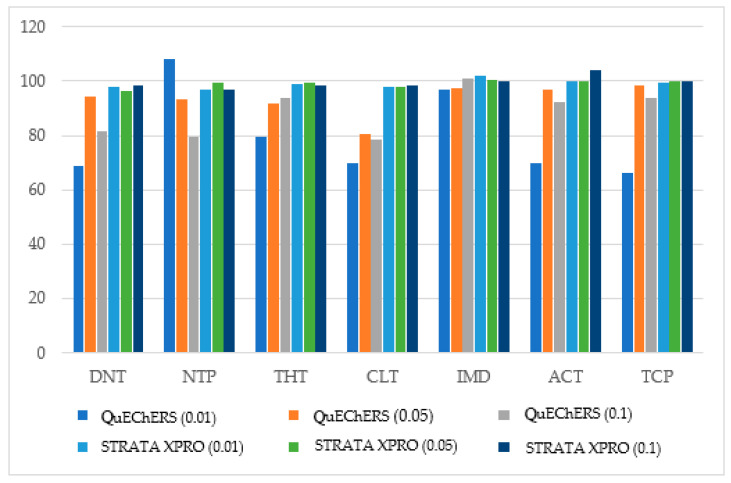
Recovery values (%) for the seven neonicotinoids (0.01, 0.05, and 0.1 mg/kg) obtained using QuEChERS (blue columns) and STRATA XPRO (red columns).

The NEOs were added to a sample without NEOs, and their recoveries were evaluated using two different extraction and cleanup procedures (QuEChERS and STRATA XPRO) to optimize the speed and recovery capacity of the NEO test. The results are reported in Figure 1.

The comparison of the results showed that the two techniques had similar efficiency for six NEOs (ACT, DTN, IMD, NTP, TCP, and THT), but the STRATA XPRO cartridge allowed a more significant recovery of CLT.

Based on this consideration and, mainly, the shorter analysis times of the STRATA XPRO cartridge compared to the QuEChERS method, STRATA XPRO was used to clean up the wheat samples from interferents in the test to be validated.

### 3.2. Method Validation

The analytical method was confirmed through twenty-seven tests. The number of tests was chosen with a balanced consideration between the number of analyses that could be performed in a single day and the expenses associated with the validation study.

#### 3.2.1. Selectivity

**Figure 2:**
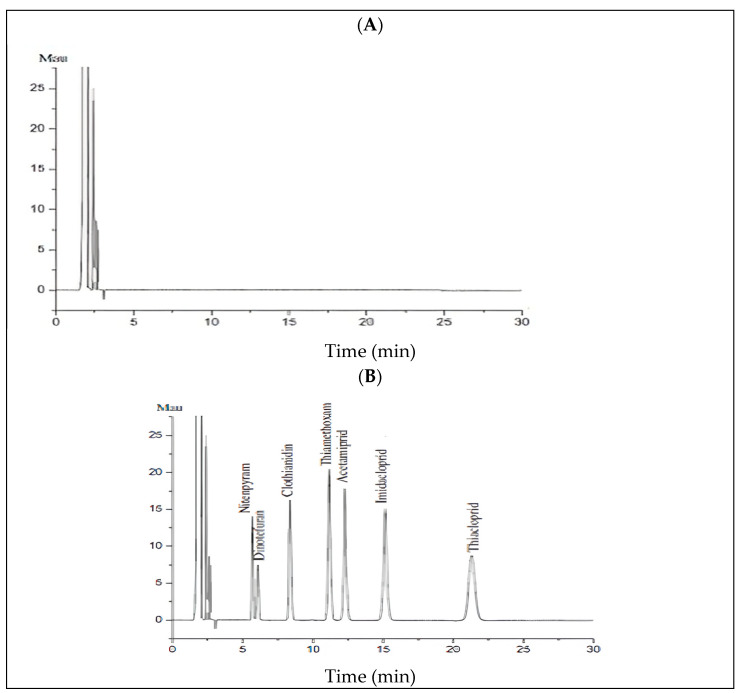
Blank’s chromatogram (**A**). Chromatogram of a sample enriched with 0.1 mg/kg of a multicomponent solution containing the seven neonicotinoids examined (**B**).

Some helpful parameters for establishing the method’s selectivity are shown in Table 2. The selectivity was evaluated by examining the consistency of NEO retention times and the absence of interferences. The absence of interferences at the retention times corresponding to the NEOs proves that the proposed extraction procedure is effective, selective, and appropriate for determining the target analytes (Figure 2).

#### 3.2.2. Linearity

The linearity of the analytical procedure was evaluated in the concentration ranges of 0.01, 0.05, and 0.1 μg/mL. The calibration curve was calculated using ordinary least-square linear regression, and the linearity was expressed by the coefficient of determination (*r*^2^). In every sample examined, the *r*^2^ value was equal to 0.999, and the equation was close to *y* = *x*. Therefore, the relationships were linear. The results are reported in Table 2.

The values of LOD and LOQ were calculated from ordinary least-squares regression data [30]. The LOD corresponds to the analyte amount for which the area is equal to 3 times the chosen standard deviation, and the LOQ corresponds to the analyte amount for which this area is equal to 10 times the standard deviation chosen [31]. The validation strategy prioritized ensures the method’s reliability and suitability for routine use, achieving extremely high performance. Consequently, the lowest limits of the validation domain, the limit of quantification (LOQ) values, were set to 0.01 mg/kg, corresponding to the maximum residue levels (MRLs) established by current European legislation; these values were chosen to quantify the undesirable presence of the selected neonicotinoids (NEOs) in wheat.

#### 3.2.3. Trueness, Precision, and Accuracy Assessment

The concentrations of the validation samples were back-calculated and used to determine the relative bias, the intra-day and inter-day precision expressed as relative standard deviation (RSD%), and the *β*-expectation tolerance intervals at the 95% probability level.

The relative standard deviation (RSD), also known as the coefficient of variation, is a measure that helps determine whether a dataset’s standard deviation is small or large relative to its mean. Essentially, the RSD provides insight into the precision of the average value of your results. The formula to calculate the RSD% is as follows:

$$\mathrm{RSD}\%=\frac{S*100}{x}$$

Tolerance intervals are statistical estimates representing a specific population percentage within a certain range of values.

Trueness and precision provide information on the systematic and random errors of the method, respectively. The excellent trueness (the relative bias (%) at each concentration level) was minimal, and good recovery values proved the high extraction efficiency of the method (Table 3). The relative standard deviations (RSD%) for the intra-day and inter-day precision, well below 5%, confirmed the NEO test’s good precision (Table 3).

**Figure 3:**
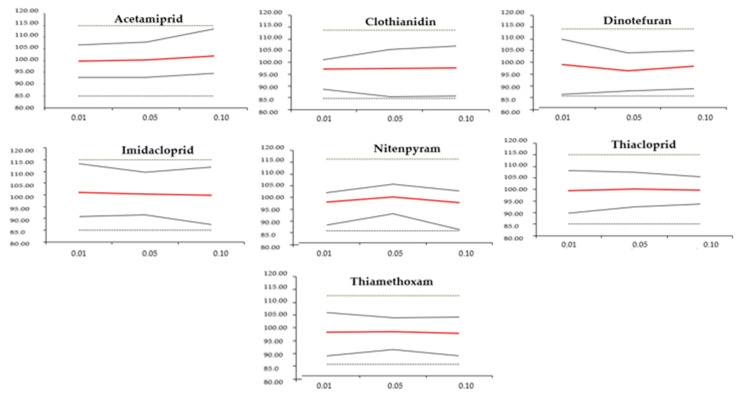
Accuracy profiles obtained for each NEO’s determination in wheat using the linear regression model. The red line is the recovery yield (%), the dotted lines are the ±15% acceptance limits, and the black lines are the upper and lower relative 95% expectation tolerance limits.

The accuracy of the proposed method was confirmed by relative upper and lower β-expectation tolerance limits (%) (Figure 3; Table 3) that remained within the acceptance thresholds for total error at every concentration level. Additionally, stringent measures were in place to ensure that future assay results did not exceed the limits of ±15% of the targeted amounts.

## 4. Discussion

Cereal crops, a primary food source globally, are frequently subjected to various pesticide treatments during their growth and post-harvest stages [32].

It is estimated that there could be a loss of about 32% in cereal production without pesticides. Hence, pesticides are crucial for maintaining a high yield [33]. However, the downside is that these pesticides leave harmful residues and can contaminate crops. Consequently, it is imperative to have suitable analytical techniques to control the pesticide residue levels in cereals. This work validated a new method to simultaneously determine seven NEOs in wheat flour. NEOs on treated plant matrices are present in small quantities and with other molecules that could interfere with their dosage. Therefore, the first analytical difficulty was searching for a cleanup system that would allow sufficient recovery for measuring and eliminating potential interfering substances. For this purpose, the performance of a system based on a novel polymer (STRATA XPRO) was juxtaposed with the QuEChERS method, which is typically employed to maximize the extraction of NEOs from food matrices. This method is known for its simplicity, efficiency, and ability. The QUECHERS method involves multiple steps: sample preparation, salting out, extraction, and cleanup by dispersive solid-phase extraction (d-SPE) using primary and secondary amine (PSA) to eliminate various polar organic acids, polar pigments, and some sugars and fatty acids. Graphitized carbon black (GCB) or C18 columns may also remove sterols, pigments, or lipids. The QuEChERS procedure consists of five steps (condition, equilibration, loading sample, washing sample, and elution of the analytes). The criticisms and limitations of the QuEChERS method are inconsistent recoveries, the incomplete removal of interferences, salt precipitation, and additional cleanup steps necessary for specific complex samples. In this work, to overcome the QuEChERS procedure’s limitations, the STRATA XPRO prepacked cartridges were tested. Strata-X PRO cartridges are prepacked with an innovative polymeric SPE sorbent with a matrix removal technology. Strata-X PRO cartridges extract analytes in only two steps (loading sample and rinse). Thus, the Strata-X PRO technology is faster than the QuEChERS test. Moreover, a single extraction step decreases the risk of losses of NEOs. Both tests demonstrated acceptable performance for studying analytes in the validation levels. Both tests gave similar recoveries for ACT, DTN, IMD, NTP, TCP, and THT. However, the STRATA test yielded the best recoveries for CLT (97.7–98.2%) compared to the QuEChERS method (69.8–80.3%). Therefore, the Strata-X PRO technology, faster and more efficient in the simultaneous recovery of the seven NEOs compared to the QuEChERS test, was used to isolate the NEOs from the wheat flour. Some considerations were made to complete the isolation. The compounds’ high polarity and low volatility influenced the selection of chromatographic method. Few studies have reported their isolation using gas chromatography (GC); the required derivatization process to increase the volatility of NEOs makes gas chromatography a second-choice method in terms of time and labor consumption [34,35]. In this work, NEOs, as in most research works which reported their isolation in plant-based food products, were quantized by high-performance liquid chromatography. The HPLC’s operational parameters were meticulously adjusted to attain the maximum possible sensitivity, precise measurements, and optimal performance in the analytic process. A Kinetex C-18 column was used to isolate NEOs. The core–shell or fine porous shell particles (sol–gel processing methods integrated with nano-structuring technology and a consistent particle size distribution) provide efficient separation and fast analytical run duration [35,36]. The core–shell technology of the Kinetex column, where a porous silica adsorbent shell envelops a solid core, enhances resolution, throughput, and sensitivity compared to other C-18 stationary phases [37]. This work used STRATA X PRO^®^ prepacked cartridges for the fast and effective extraction of natural extractable organic materials (NEOs) from wheat. After the extraction, a reverse-phase high-performance liquid chromatography with diode array detection (HPLC-DAD) analysis could be conducted to accurately measure the dosage of the NEOs. Its versatility, precision, and relatively low cost suggest a possible use in routine NEO residue analysis. The DAD was chosen as a detector of the chromatographic apparatus because NEOs absorb in the UV spectrum, and the DAD allows the recording of spectra at different wavelengths [38,39]. Furthermore, we chose the DAD instead of more expensive detectors, such as a mass spectrometer, as a DAD detector is often available in analysis laboratories that carry out the analysis of NEOs. Concerning the mobile phase, a solvent gradient optimized to achieve a brief run time, a uniform distribution of analytes within the elution window, and adequate retention (all analytes eluted within a time window of twenty-two minutes) were used. Finally, a fast and economic method to determine NEOs in wheat was validated, according to the strategy proposed by the Commission of the Société Française des Sciences et Techniques Pharmaceutiques (SFSTP), based on the accuracy profile approach, to ensure data traceability, prevent inaccurate measurements, and, in general, evaluate the method’s performance as a routine food control technique. 

The validation proved that the proposed method enjoyed a robust linear relationship between observed and predicted values since its coefficients of determination exceeded 0.999 within all the concentration ranges tested (0.01, 0.05, and 0.1 μg/mL) for all pesticides. The analytical results for each pesticide in both solvent and matrix demonstrated that the matrix effect was negligible in determining the target compounds (Table 2). The relative bias (%) shown in Table 3 indicated excellent trueness at each concentration level, i.e., the absence of systematic and random errors. The elevated recovery showed the important extraction efficiency of the method. The relative standard deviations (RSDs%) for intra-day and inter-day measurements demonstrated reliable precision. Finally, the relative upper and lower β-expectation tolerance limits (%) that did not exceed the acceptance limits for total error at each concentration level confirmed that the risk of future assay results deviating by more than ±15% from the targeted amounts was strictly controlled.

## 5. Conclusions

For the first time, this work developed and validated a protocol to measure seven nicotinoids (acetamiprid, clothianidin, dinotefuran, imidacloprid, nitenpyram, thiacloprid, and thiamethoxan) in wheat at the same time. The experimental protocol employed STRATA X PRO^®^ prepacked cartridges to ensure an efficient and quick NEO extraction from wheat and a reverse-phase HPLC-DAD analysis to measure them. The NEOs’ recoveries between 70 and 110%, the regression coefficient close to one, the limit of quantification above the maximum concentration of NEOs to be determined, intra- and inter-day measurements with an accuracy lower than 10% capable of satisfying the requirements of the European Commission, and the true value that lies around the measured result with a confidence level of 95% validated the NEO HPLC method proposed.

The method’s low cost (reduced usage of organic solvents due to the use of SPE cartridges) and fast and easy operation suggest the use of the validated experimental protocol in routine analyses to measure acetamiprid, clothianidin, dinotefuran, imidacloprid, nitenpyram, thiacloprid, and thiamethoxan in wheat and other food matrices. The process of validation is costly and time-intensive. The scientific community should intensify its efforts to validate analytical methods to pave the way for precise, accurate, and selective tests that offer reliable and consistent results under varying conditions and guarantee food quality.

## Figures and Tables

**Table 1:** NEOs’ chromatographic and spectroscopic identification parameters.

NEOs	λ_max_ (nm)	Rt	UV Spectrum
**Acetamiprid** 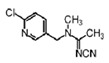	**245**	**12.5**	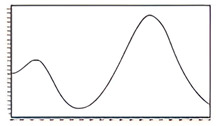
**Imidacloprid** 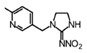	**270**	**15.1**	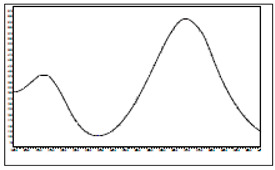
**Nitenpyram** 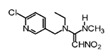	**270**	**6.2**	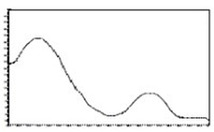
**Thiacloprid** 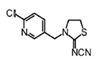	**245**	**22.0**	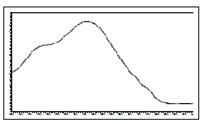
**Clothianidin** 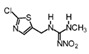	**270**	**7.9**	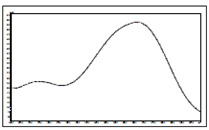
**Thiamethoxam** 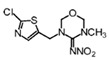	**255**	**11.3**	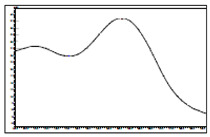
**Dinotefuran** 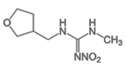	**270**	**6.4**	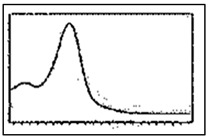

**Table 2:** Analytical chromatographic validation parameters obtained from the calibration curve (*k* = 3; *n* = 3; *m* = 3).

NEOs		Slope	Intercept	r^2^	LOQ (mg/kg)	LOD (mg/kg)
**DNT**	Solvent Matrix	153.24 155.12	0.68 0.47	0.9996 0.9991	0.01	0.003
**NTP**	Solvent Matrix	201.76 203.95	0.20 0.04	0.9997 0.9990	0.01	0.003
**THT**	Solvent Matrix	148.82 149.94	0.20 0.04	0.9998 0.9999	0.01	0.003
**CLT**	Solvent Matrix	171.43 169.82	−0.44 −0.03	0.9999 0.9998	0.01	0.003
**IMD**	Solvent Matrix	191.57 187.05	0.59 0.93	0.9992 0.9992	0.01	0.003
**ACT**	Solvent Matrix	214.56 218.85	0.68 0.47	0.9995 0.9990	0.01	0.003
**TCP**	Solvent Matrix	176.86 175.50	−0.44 −0.03	0.9997 0.9993	0.05	0.02

**Table 3:** Validation parameters results for the selected NEOs in wheat (*k* = 3; *n* = 3; *m* = 3).

NEOs	*Intra-Day*			*Inter-Day*			*Relative Bias*			*β*-Expectation		
	RSD (%)			RSD (%)			(%)			Tolerance Limit (%)		
	mg/kg			mg/kg			mg/kg			mg/kg		
	0.01	0.05	0.1	0.01	0.05	0.1	0.01	0.05	0.1	0.01	0.05	0.1
**DNT**	2.1	2.0	1.3	1.7	1.9	1.9	−1.0	−3.8	−1.7	[11.2; −13.2] [8.1; −15.7] [7.0; −10.4]		
**NTP**	1.2	1.3	1.4	0.3	0.5	0.6	−2.9	−0.8	3.2	[3.9; −9.7] [5.3; −6.9] [4.8; 11.2]		
**THT**	1.6	1.1	1.2	0.5	1.2	1.1	1.0	0.8	1.4	[8.4; −10.4] [6.2; −7.8] [7.0; −9.8]		
**CLT**	0.9	1.4	1.5	0.6	1.9	2.2	2.3	2.0	1.8	[4.2; −8.8] [8.3; −12.3] [9.7; 13.3]		
**IMD**	1.5	1.2	1.7	2.1	1.8	2.0	1.0	0.3	0.2	[12.2; −10.2 ] [9.4; −8.8] [12.0; −12.4]		
**ACT**	0.6	0.9	1.2	2.2	1.6	1.9	0.1	0.2	2.0	[6.7; −6.9] [7.6; −7.2] [11.5; −7.5]		
**TCP**	1.3	0.7	0.9	1.4	0.9	0.8	0.5	0.1	0.2	[8.8; −9.8] [7.4; −7.6] [5.8; −6.2]		

## Data Availability

The original contributions presented in the study are included in the article, further inquiries can be directed to the corresponding author.
